# Author Correction: Yolk sac macrophage progenitors traffic to the embryo during defined stages of development

**DOI:** 10.1038/s41467-018-06065-9

**Published:** 2018-09-07

**Authors:** C. Stremmel, R. Schuchert, F. Wagner, R. Thaler, T. Weinberger, R. Pick, E. Mass, H. C. Ishikawa-Ankerhold, A. Margraf, S. Hutter, R. Vagnozzi, S. Klapproth, J. Frampton, S. Yona, C. Scheiermann, J. D. Molkentin, U. Jeschke, M. Moser, M. Sperandio, S. Massberg, F. Geissmann, C. Schulz

**Affiliations:** 1 Medizinische Klinik und Poliklinik I, Klinikum der Universität, Ludwig-Maximilians-Universität, Marchioninistrasse 15, 81377 Munich, Germany; 2 Walter-Brendel-Center for Experimental Medicine, Ludwig-Maximilians-Universität, Marchioninistrasse 15, 81377 Munich, Germany; 3 Immunology Program, Sloan Kettering Institute, Memorial Sloan Kettering Cancer Center, 1275 York Avenue, New York, NY 10065 USA; 4 Developmental Biology of the Innate Immune System, LIMES-Institute, University of Bonn, Carl-Troll-Straße 31, 53115 Bonn, Germany; 5 Klinik und Poliklinik für Frauenheilkunde und Geburtshilfe, Klinikum der Universität, Ludwig-Maximilians-Universität, Maistrasse 11, 80337 Munich, Germany; 6 Department of Pediatrics, Cincinnati Children’s Hospital Medical Center, 3333 Burnet Avenue, Cincinnati, OH 45229 USA; 7 Department of Molecular Medicine, Max Planck Institute of Biochemistry, Am Klopferspitz 18, 82152 Martinsried, Germany; 8 Institute of Cancer and Genomic Sciences, College of Medical and Dental Sciences, University of Birmingham, Edgbaston, Birmingham B15 2TT UK; 9 Department of Immunology, The Weizmann Institute of Science, 234 Herzl Street, Rehovot 76100, Israel; 10 Howard Hughes Medical Institute, Cincinnati Children’s Hospital Medical Center, 3333 Burnet Avenue, Cincinnati, OH 45229 USA; 11 DZHK (German Center for Cardiovascular Research), Partner Site Munich Heart Alliance, Biedersteiner Strasse 29, 80802 Munich, Germany

Correction to: *Nature Communications*; 10.1038/s41467-017-02492-2; published online 8 Jan 2018.

This article contains errors in Figs. 5 and 6, for which we apologize. In Fig. 5f, the image ‘E12.5 tail’ was inadvertently replaced with a duplicate of the image ‘E12.5 trunk’ from the same panel. In Fig. 6d, the image ‘E9.5/OH-TAM E8.5, embryo’ was inadvertently replaced with a duplicate of the image ‘E10.5/ OH-TAM E8.5, embryo’ from Fig. 6b. The corrected versions of these figures appear below as Figs. 1 and 2. The raw data associated with these experiments can be accessed via the following link: https://figshare.com/articles/Yolk_sac_macrophage_progenitors_traffic_to_the_embryo_during_defined_stages_of_development/6930629.

**Fig. 1:**
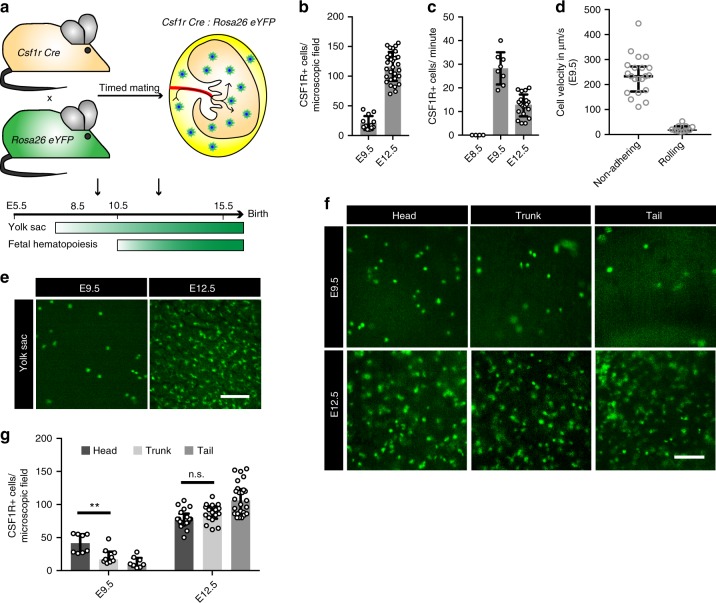
▓

**Fig. 2:**
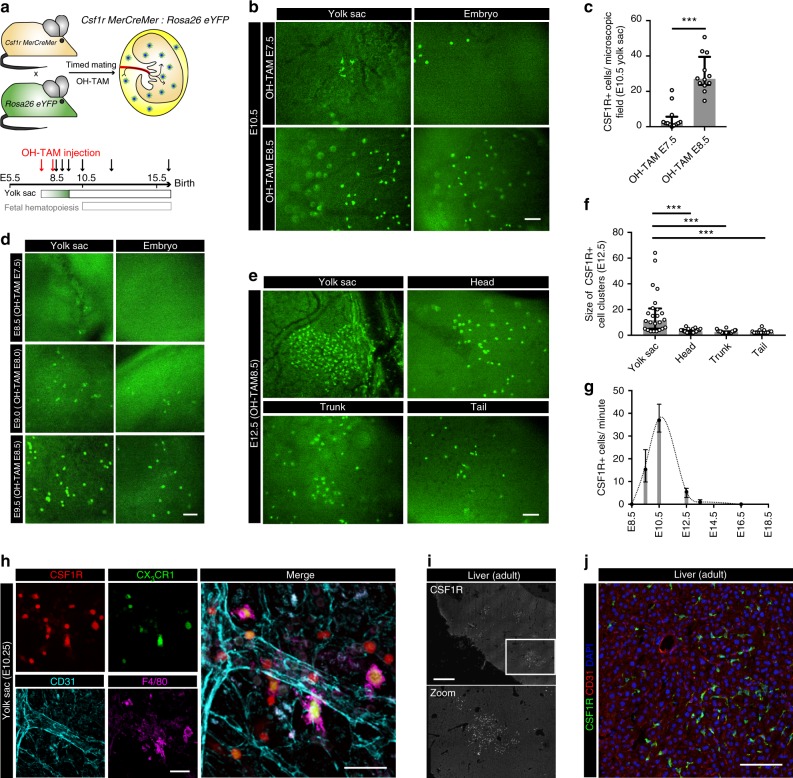
▓

