# Supplementary material for: Yolk sac macrophage progenitors traffic to the embryo during defined stages of development
Source: Nat Commun. 2018 Jan 8;9:75. doi: 10.1038/s41467-017-02492-2 (PMC5758709; doi:10.1038/s41467-017-02492-2)
Supplement: Supplementary file 1 — Supplementary Information [file 41467_2017_2492_MOESM1_ESM.pdf]

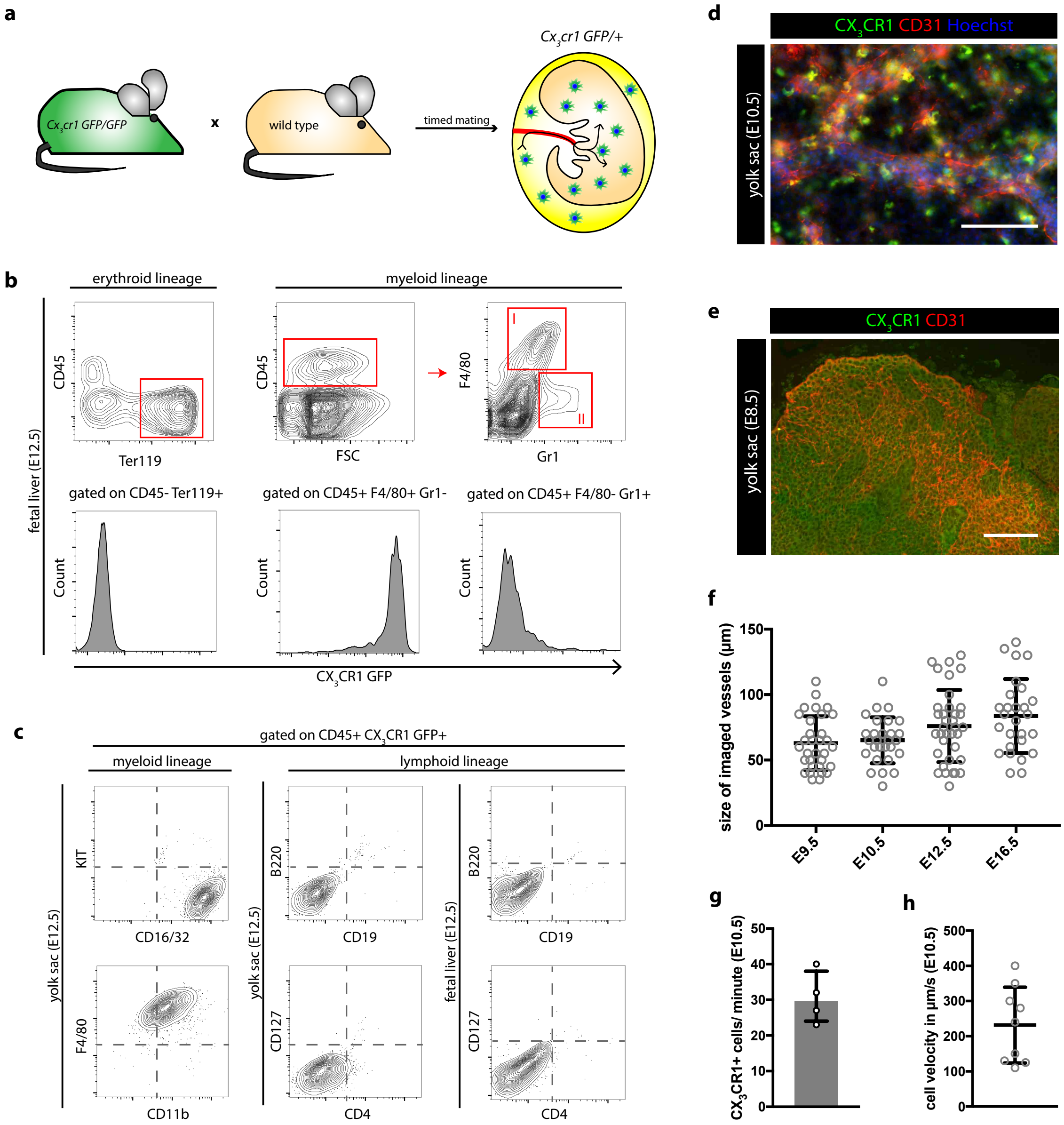

Supplementary Figure 1: *Cx₃cr1$^{GFP/+}$* reporter effectively labels YS pre-macrophages. (a) Schematic graph of the *Cx₃cr1$^{GFP/+}$* mouse model used for embryonic analyses. (b, c) Flow cytometry analyses of fetal liver and YS cells at E12.5 as indicated. (b) Quantification of CX_3CR1 GFP+ cells in the erythroid (Ter119+) and myeloid lineage; CD45+ F4/80+ Gr1- macrophages (population I) and CD45+ F4/80- Gr1+ neutrophils (population II). (c) Surface marker profile of CD45+ CX_3CR1 GFP+ cells screened for KIT, CD16/32, F4/80 and CD11b (myeloid markers) as well as B220, CD19, CD127 and CD4 (lymphoid markers). Flow cytometry plots (b, c) show an individual representative experiment. At least 3 embryos of minimum 2 independent litters were analyzed per time point. (d, e) YS whole mount staining of a *Cx₃cr1$^{GFP/+}$* YS (green) at E10.5 (d) and E8.5 (e). Endothelium was stained for CD31 (red). Nuclei were visualized by Hoechst (d). (f) Quantification of YS vessel size used for trafficking quantifications at indicated time points; mean ± SD. (g, h) Quantification of trafficking kinetics in connection to maternal circulation at E10.5. (g) Quantification of intravascular CX_3CR1+ cells in an average sized vessel; median ± IQR. (h) Velocity of flowing CX_3CR1+ cells in the YS vasculature. Scale bars are 100 µm (d, e).

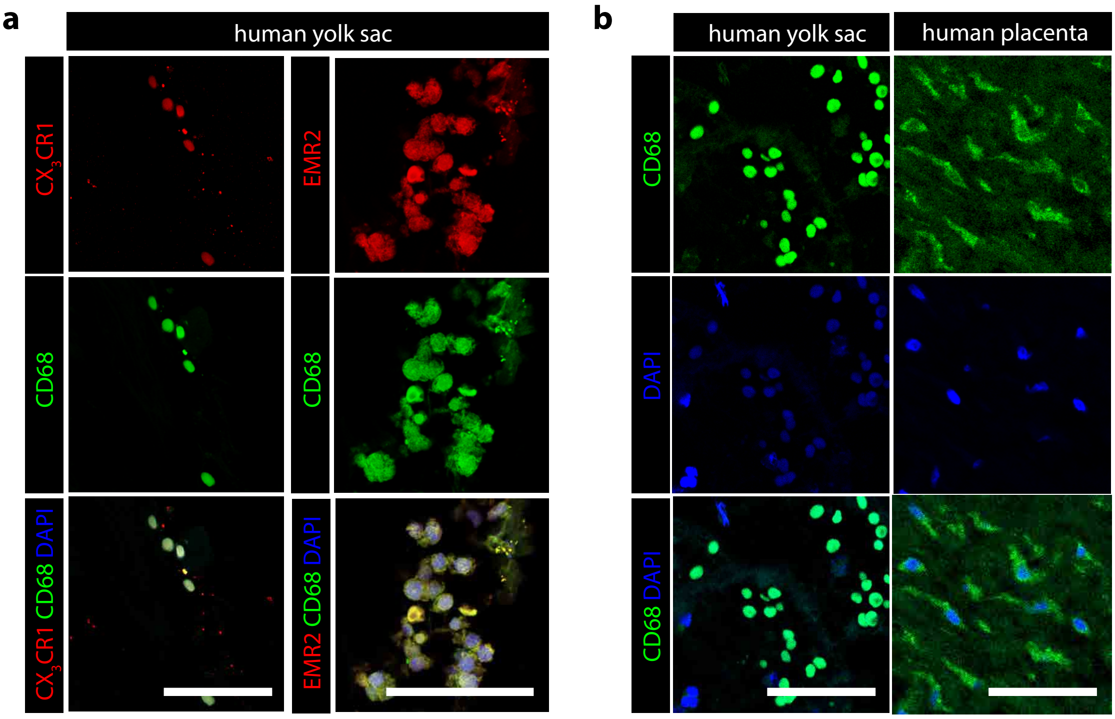

Supplementary Figure 2: Human YS macrophages. (a, b) Confocal fluorescence images of human YS and placenta stained for CD68 (green), CX_3CR1 (red, upper left panel in (a)), EMR2 (upper right panel in (a)) and DAPI (blue). Samples were obtained from abortion material, age of gestation: 9 weeks + 1 day. Scale bars are 50 µm.

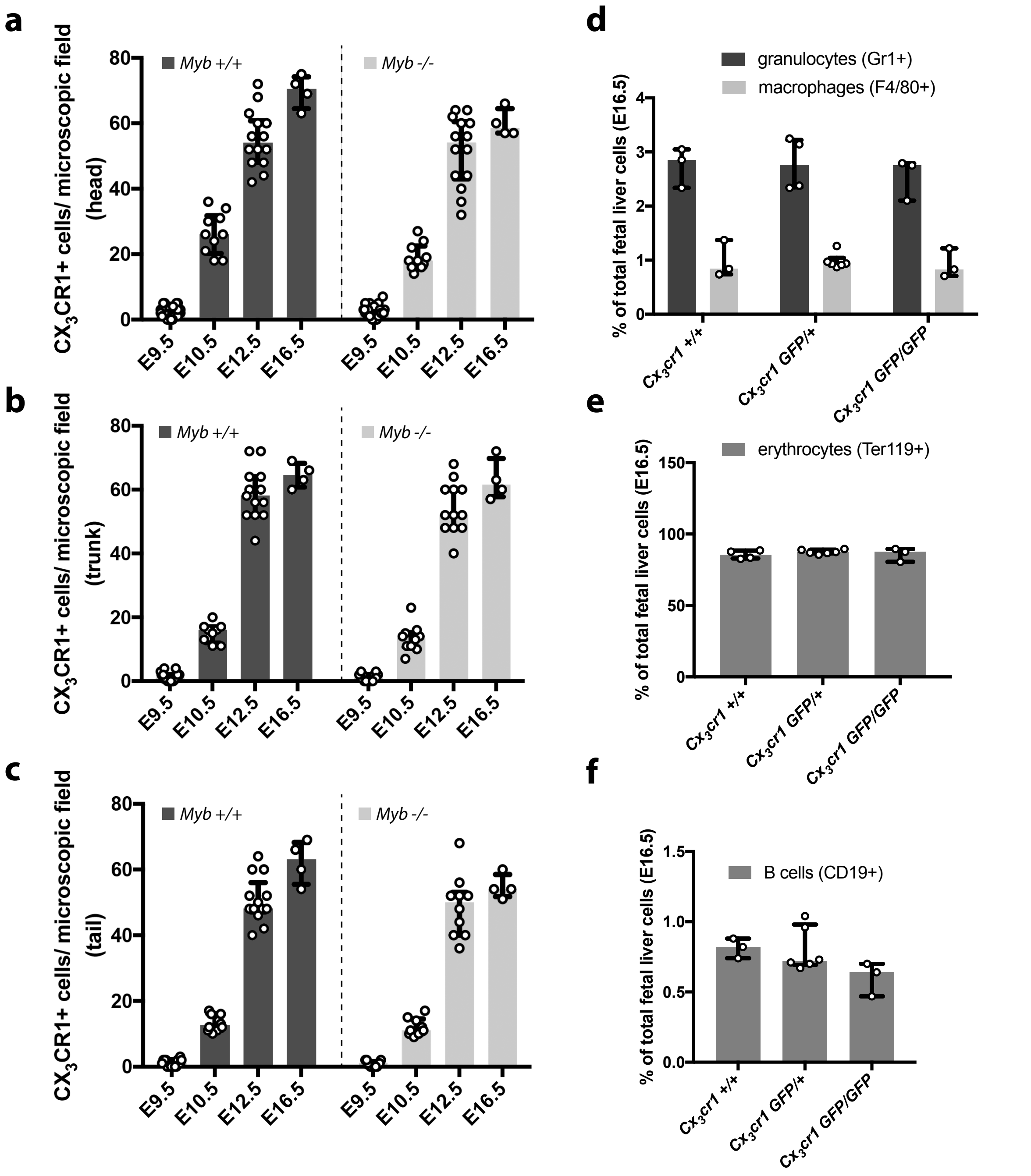

Supplementary Figure 3: CX_3CR1+ cells in the absence of MYB and CX_3CR1. (a-c) Quantification of CX_3CR1+ cells per microscopic field of 400 µm x 400 µm in *Myb*$^{-/-}$ and *Myb*$^{+/+}$ mice in indicated embryonic regions at indicated time points. (d-f) Quantification of different cell lines by indicated markers in the E16.5 fetal liver of *Cx*$_3$*cr1*$^{+/+}$, *Cx*$_3$*cr1*$^{GFP/+}$ and *Cx*$_3$*cr1*$^{GFP/GFP}$ mice. In all individual panels, comparisons between different genotypes did not show significant differences (Kruskal-Wallis test: p=0.6129 (d), p=0.8370 (e), p=0.0553 (f)). All graphs show median ± IQR.

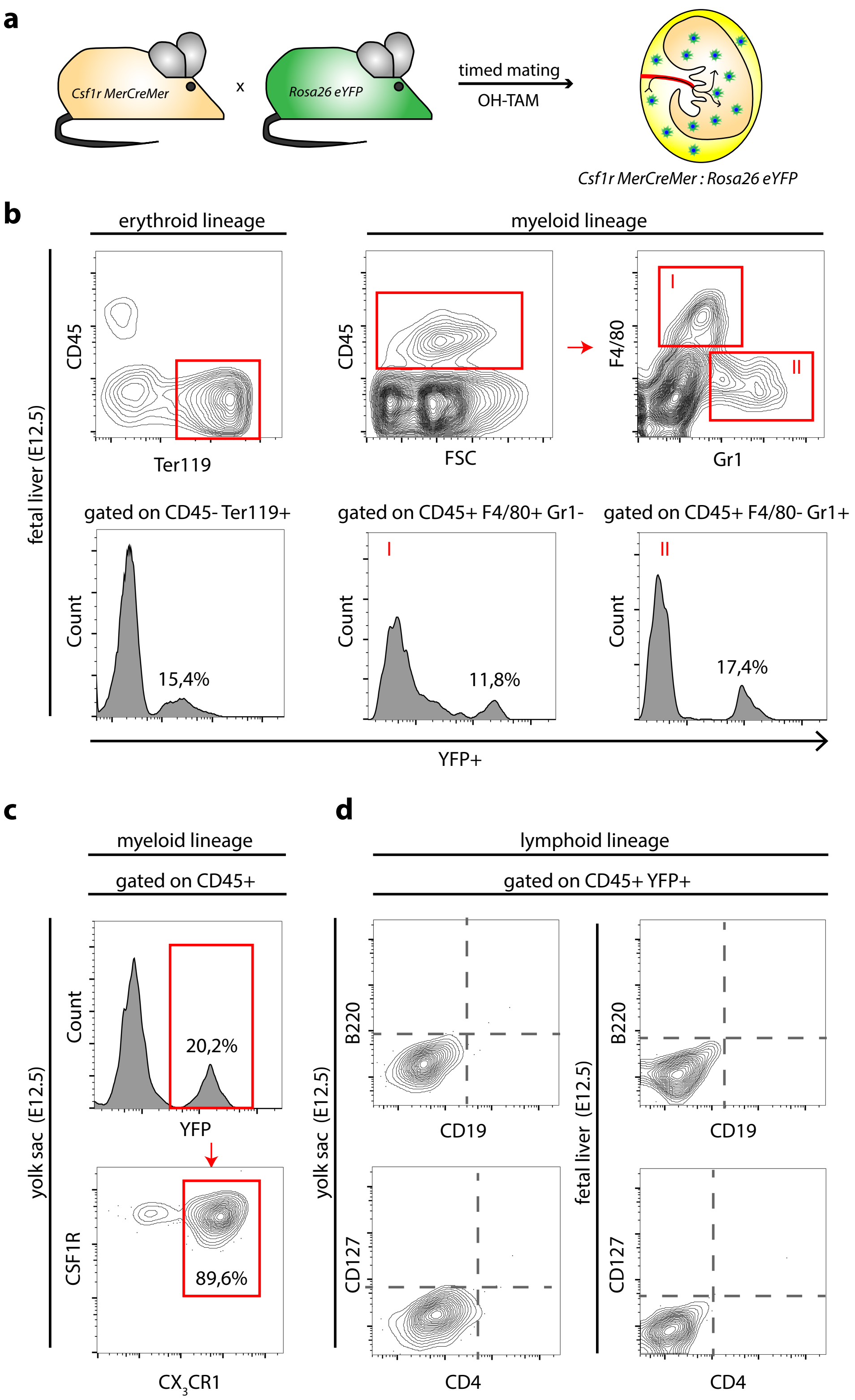

Supplementary Figure 4: CSF1R pulse labeling model tracks EMPs. (a) Schematic graph for the *Csf1r*^{MerCreMer}*:Rosa26*^{eYFP} mouse model with OH-TAM pulse labeling at E8.5. (b) Analysis of labeling efficiency of erythroid and myeloid lineages in E12.5 fetal liver. (c-d) Flow cytometry analysis of *Csf1r*^{MerCreMer}*:Rosa26*^{eYFP} pulse-labeled cells. (c) Quantification of CX_3CR1-expressing cells among YFP+ cells in the YS at E10.5. (d) Surface expression of indicated lymphoid markers at E12.5 in the YS and fetal liver. Flow cytometry plots show an individual representative experiment. At least 3 embryos of minimum 2 independent litters were analyzed per time point.

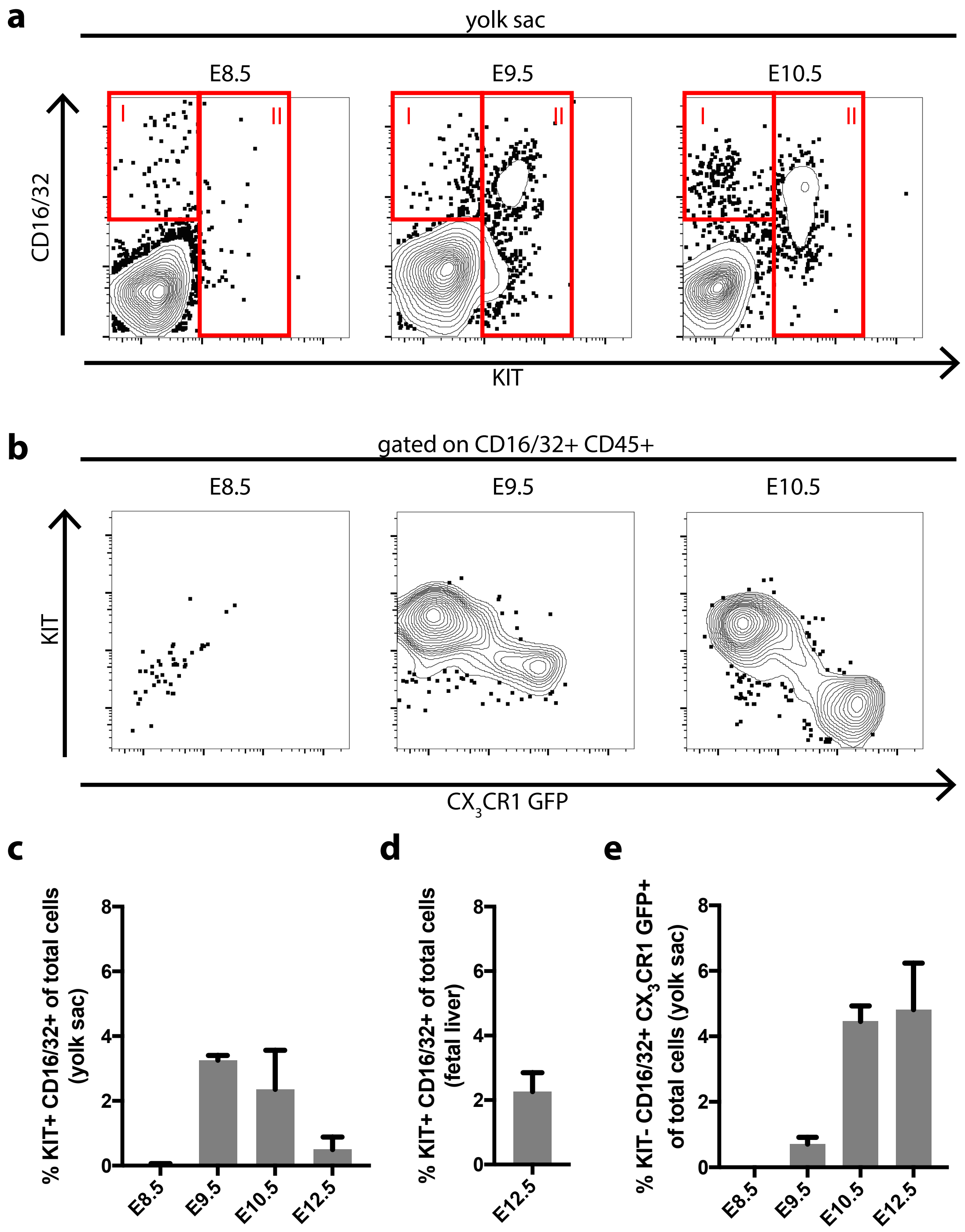

Supplementary Figure 5: Primitive YS macrophages appear before the first detection of KIT+ cells. (a) Flow cytometry plots of CD16/32+ KIT- (population I) and KIT+ (population II) cells in the embryonic YS at indicated time points. (b-e) Plots (b) and corresponding quantifications (c-e) of KIT+ CD16/32+ progenitors in the YS (c) and fetal liver (d) as well as KIT- CD16/32+ CX_3CR1 GFP+ cells in the YS (e) at indicated time points. Plots show an individual representative experiment. Bar graphs (c-e) show mean ± SD. At least 3 embryos of minimum 2 independent litters were analyzed per time point.
