# Supplementary material for: Yolk sac macrophage progenitors traffic to the embryo during defined stages of development
Source: Nat Commun. 2018 Jan 8;9:75. doi: 10.1038/s41467-017-02492-2 (PMC5758709; doi:10.1038/s41467-017-02492-2)
Supplement: Supplementary file 3 — Description of Additional Supplementary Files [file 41467_2017_2492_MOESM3_ESM.pdf]

Description of Additional Supplementary Files

File Name: Supplementary Movie 1
Description: **CX_3CR1+ pre-macrophages infiltrate the YS vasculature.** Intravital microscopy of the YS at E10.5 showing a CX_3CR1+ cell entering the blood stream. Scale bar is 100 µm

File Name: Supplementary Movie 2
Description: **Temporal re-adhesion of CX_3CR1+ pre-macrophages after vascular infiltration.** Intravital microscopy of the YS at E10.5 showing temporal readhesion of a CX_3CR1+ cell after vascular infiltration. Scale bar is 100 µm.

File Name: Supplementary Movie 3
Description: **Trafficking of CX_3CR1+ cells in the YS at E10.5.** Intravital microscopy of CX_3CR1+ cells in the YS at E10.5. Scale bar is 100 µm.

File Name: Supplementary Movie 4
Description: **Trafficking of CX_3CR1+ cells in the YS at E12.5**. Intravital microscopy of CX_3CR1+ cells in the YS at E12.5. Scale bar is 100 µm.

File Name: Supplementary Movie 5
Description: **Trafficking of CX_3CR1+ cells in the YS at E16.5.** Intravital microscopy of CX_3CR1+ cells in the YS at E16.5. Scale bar is 100 µm.

File Name: Supplementary Movie 6
Description: **Pre-macrophage adhesion in the embryonic head region.** Intravital microscopy of CX_3CR1+ cells in the embryonic head region at E10.5. Adhering cell is indicated by an arrow head. Scale bar is 100 µm.

File Name: Supplementary Movie 7
Description: **High resolution microscopy of pre-macrophage trafficking in the YS.** Intravital spinning disc confocal microscopy of CX_3CR1+ cells (green) in the YS of Cx_3cr1^{Cre}:$Rosa26^{mT/mG}$ mice at E10.5. Scale bar is 20 µm.

File Name: Supplementary Movie 8
Description: **Intravascular protrusions of mature macrophages.** Intravital spinning disc confocal microscopy of a CX_3CR1+ macrophage (green) in the endothelium (red) of a YS vessel in Cx_3cr1^{Cre}:$Rosa26^{mT/mG}$ mice at E10.5. Scale bar is 20 µm.

File Name: Supplementary Movie 9
Description: **Pre-macrophages in the YS of *Myb*$^{-/-}$ mice on E10.5.** Intravital microscopy of CX_3CR1+ cells in the YS of $Cx_3cr1^{GFP/+}$:*Myb*$^{-/-}$ mice at E10.5. Scale bar is 100 µm.

File Name: Supplementary Movie 10
Description: **Pre-macrophages in the YS of *Myb*$^{-/-}$ mice on E12.5.** Intravital microscopy of CX_3CR1+ cells in the YS of $Cx_3cr1^{GFP/+}$:*Myb*$^{-/-}$ mice at E12.5. Scale bar is 100 µm.

File Name: Supplementary Movie 11
Description: **CSF1R+ cells in the YS at E9.5.** Intravital microscopy of the YS in *Csf1r*^{Cre}*:Rosa26*^{eYFP} mice at E9.5. Scale bar is 100 µm.

File Name: Supplementary Movie 12
Description: **CSF1R+ cells in the YS at E12.5.** Intravital microscopy of the YS in *Csf1r*^{Cre}*:Rosa26*^{eYFP} mice at E12.5. Scale bar is 100 µm.

File Name: Supplementary Movie 13
Description: **Pulse-labeled CSF1R+ cells in the YS at E10.5.** Movie of an embryonic YS at E10.5 after OH-TAM-based pulse labeling at E8.5 in *Csf1r*^{MerCreMer}*:Rosa26*^{eYFP} mice. Scale bar is 100 µm.

File Name: Supplementary Movie 14
Description: **Pulse-labeled CSF1R+ cells in the YS at E12.5**. Movie of an embryonic YS at E12.5 after OH-TAM-based pulse labeling at E8.5 in *Csf1r*^{MerCreMer}*:Rosa26*^{eYFP} mice. Scale bar is 100 µm.

File Name: Supplementary Movie 15
Description: **Pulse-labeled KIT+ EMPs in the YS.** Movie of an embryonic YS at E10.5 after OH-TAM-based pulse labeling at E8.5 in *Kit*^{MerCreMer}*:Rosa26*^{eYFP} mice. Scale bar is 100 µm.
